# Urban drinking and driving: comparison of electric scooter and bicycle related accidents in facial fracture patients

**DOI:** 10.4317/medoral.25662

**Published:** 2022-10-16

**Authors:** Olli-Jussi Murros, Tero Puolakkainen, Anne Abio, Hanna Thorén, Johanna Snäll

**Affiliations:** 1 Department of Oral and Maxillofacial Diseases, University of Helsinki and Helsinki University Hospital, Finland; 2 Injury Epidemiology and Prevention Research Group, Turku Brain Injury Centre, Division of Clinical Neurosciences, Turku University Hospital and University of Turku, Turku, Finland; 3 Heidelberg Institute of Global Health, University of Heidelberg, Heidelberg, Germany; 4 Department of Oral and Maxillofacial Surgery, Institute of Dentistry, University of Turku, Turku, Finland; 5 Department of Oral and Maxillofacial Diseases, Turku University Hospital, Turku, Finland

## Abstract

**Background:**

In recent years, electric scooters (e-scooter) have emerged as an alternative mode of urban transport due to their availability and effortless use. However, e-scooter-related trauma and injuries, especially to the head, have received wide media coverage and raised public concern about their safety. We aim to determine and compare clinically relevant variables, incidence, and severity between bicycle and e-scooter-related facial fractures and potential protective measures for injury prevention.

**Material and Methods:**

This retrospective study comprised all patients admitted to a tertiary trauma center with bicycle or e-scooter-related facial fractures between January 2019 and October 2020. Patient- and injury-related variables, including demographics, injury mechanisms, helmet use, influence of alcohol, types of facial injuries, types of other injuries, given treatment, and hospital stay, were collected, analysed, and compared between bicycle and e-scooter injuries.

**Results:**

Altogether 169 patients with facial fractures, 124 bicycle-related injuries (73.4%) and 45 e-scooter-related injuries (26.6%) were included. Alcohol involvement was significantly higher in e-scooter patients (88.9%) than in bicycle patients (31.5%) (*p*<0.001). Driving under the influence of alcohol was associated with driving without a helmet in both groups (*p*<0.001). In multivariate analyses, e-scooter accidents were 18 times more likely to occur under the influence of alcohol (OR 17.85, *p*<0.001) and were more likely to involve collision with a stationary object (OR 3.81, *p*=0.028). E-scooter patients were significantly younger (OR 0.95, *p*<0.001) and had significantly more cranial fractures (OR 10.15, *p*=0.014) than bicycle patients.

**Conclusions:**

Compared with patients in bicycle accidents, facial fracture patients injured in e-scooter accidents are younger, are more likely under the influence of alcohol, and sustain more severe craniofacial skeleton fractures. Our results for both groups of patients advocate stricter adherence to helmet and road safety legislation as well as public education for injury prevention.

** Key words:**Electric scooter, bicycle, facial injuries, helmet.

## Introduction

During the past decade the electric scooter (e-scooter) has emerged as a competitive alternative transportation mode to the bicycle. The founding of numerous commercial e-scooter rental companies has made this mode of transport widely available and accessible to consumers. However, unenforced alcohol and helmet regulations in addition to non-compliance with safety regulations has led to increased e-scooter-related trauma and emergency care admissions globally (1-3).

According to a recent Finnish observational study, the incidence of e-scooter accident-related emergency department (ED) admissions is 18.0 per 100 000 rides (4). There are multiple factors underlying this high incidence, but a noTable contributing factor is that almost half of the e-scooter rides occur against regulations (5). Compared with bicycle accidents, e-scooter riders are four times more likely to be under the influence of alcohol and significantly less likely to be wearing a helmet (6,7). Alcohol seems to be a particular risk factor for head injuries since it has been shown that patients with e-scooter-related craniomaxillofacial trauma are ten times more likely to be under the influence of alcohol than patients without craniomaxillofacial injuries (8).

E-scooter-related injuries vary from superficial soft tissue lacerations to severe, permanently disabling and potentially fatal injuries, and they are frequently associated with craniofacial injuries (9-11). Indeed, the annual incidence of e-scooter-related craniofacial injuries has tripled in the last decade (1). The incidence of facial fractures in e-scooter accidents has been reported to be around 5% (1,11). Specifically, the most common facial fracture locations involve the upper face and midface (12,13). However, it has also been shown that almost 40% of patients with e-scooter-related craniofacial trauma have closed head injuries and that skull fractures are the most common type of craniofacial fracture (1). This might give an indication of the characteristic injury mechanisms and circumstances of e-scooter accidents such as the attempt of e-scooter riders to break their forward fall and the lack of helmet use. This also highlights the need for emergency care providers to be aware of closed head injuries as associated injuries while evaluating patients with e-scooter-related facial injuries.

There is a paucity of studies with comparisons of clinical and demographic variables between bicycle- and e-scooter-related facial fracture patients (14). As the number of e-scooter-related injuries is expected to increase in the future, we aimed to clarify differences between the injury profiles related to these modes of transport. We hypothesized that the factors leading to injury differ between vehicles, so a more precise approach to the underlying causes of accidents could provide a better understanding of methods for injury prevention.

## Material and Methods

- Study design

This retrospective study comprised all patients admitted to a tertiary trauma centre (Helsinki University Hospital, Helsinki, Finland) or Helsinki University Children’s Hospital with any type of facial fracture induced by bicycle or e-scooter trauma between January 2019 and October 2020. Data were retrieved from electronic patient records according to predetermined variables for all patients with a radiologically confirmed facial fracture.

- Study variables

Injury-related variables were compared with respect to the type of vehicle used (i.e. bicycle or e-scooter).

The main injury-related variable was being under the influence of alcohol at the time of injury, which was categorized dichotomously. Alcohol involvement was confirmed with a blood test, breathalyser, or information provided by the patient or the paramedics.

Other injury-related variables were the following: specific injury mechanism, helmet use, primary intubation, presence of associated injury (AI), number of AIs (one or two or more), subtype of AI, need for intensive care unit (ICU) treatment, and length of hospitalization (days).

Injury mechanism was divided into four groups: 1) falling over, 2) collision with a stationary object, 3) collision with a motorized vehicle, and 4) collision with a pedestrian or non-motorized vehicle. AIs were defined as noTable injuries outside the facial skeleton. Superficial soft tissue injuries, ligament injuries, ocular injuries, and brain concussions were excluded. AIs were further categorized into cranial fracture, intracranial haemorrhage, upper limb injury, thoracic and abdominal injury, blunt cerebrovascular injury (BCVI), cervical spine injury, pelvic injury, and lower limb injury.

Explanatory variables were age and sex.

In addition, differences between bicycle and e-scooter accidents regarding time of day, weekday and month, specific facial injury types, and need for surgical treatment for facial fracture were reported. Isolated, unilateral zygomatic-maxillary and/or orbital fractures were grouped as zygomatic-maxillary-orbital (ZMO) fractures. Le Fort fractures and other different combinations of midfacial fractures were classified as combined midfacial fractures. Upper facial fractures included fractures of the superior wall of frontal sinus and upper orbital rims. Simultaneous fractures to at least two different levels of the facial skeleton (mandible, mid-face, upper third) were reported as combination fractures of facial thirds.

- Statistical analyses

Descriptive statistics were presented as percentage and number for categorical variables. By contrast, continuous variables were presented as mean and standard deviation if normally distributed, and as median and interquartile range if not normally distributed. The cross-tabulations were conducted with Pearson Chi-Square tests and/or Fisher’s Exact test if a cell had five or fewer observations. Firth logistic regression analysis was conducted due to the small dataset. The Firth logistic regression method reduces bias in generalized linear models by penalizing the maximum likelihood estimates. Univariate models were conducted for all variables, with the type of vehicle considered as the outcome (coded as 0 for bicycle and 1 for e-scooter). The multivariable model was determined based on variables in the univariate model with p≤0.2. The Variance Inflation Factor (VIF) was used to test for multicollinearity in the final model. All predictor/explanatory variables were found to have VIF values of less than 4, hence, collinearity was minimal in the final model. Data analysis was conducted using Stata 17 (StataCorp, TX, USA). Statistical significance was set at *p*<0.05, and 95% confidence intervals are presented.

## Results

**Figure 1:**
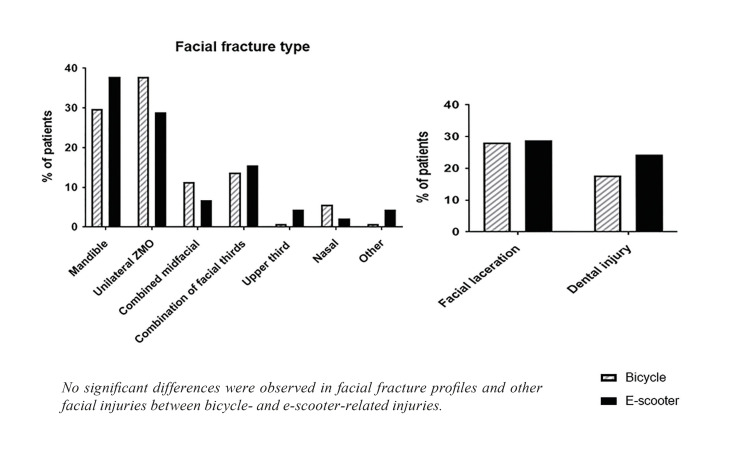
Specific facial injury profiles for bicycle- and e-scooter- related injuries.

Altogether, 169 patients were included in the study of which 124 were injured in bicycle accidents and 45 in e-scooter accidents. Mandibular (bicycle *n*=37, 29.8%, e-scooter *n*=17, 37.8%) and unilateral ZMO (bicycle *n*=47, 37.9%, e-scooter *n*=13, 28.9%) fractures were the most common facial fractures in both study populations (Fig. 1) without statistically significant differences. Regarding other facial injuries, the occurrence of facial lacerations (bicycle *n*=35, 28,2%, e-scooter *n*=13, 28.9%) and dental injuries (bicycle *n*=22, 17.7%, e-scooter *n*=11, 24.4%) was similar in both groups.

Consumption of alcohol was common as 46.7% of patients had consumed alcohol before the injury. Driving without a helmet was associated significantly with alcohol use (*p*<0.001) (Table 1). Patients who were under the influence of alcohol were more likely to sustain injury in collision with a stationary object (*p*=0.026), whereas collision with a motorized vehicle was only found in patients who were driving without alcohol use (*p*=0.004).

**Table 1:**
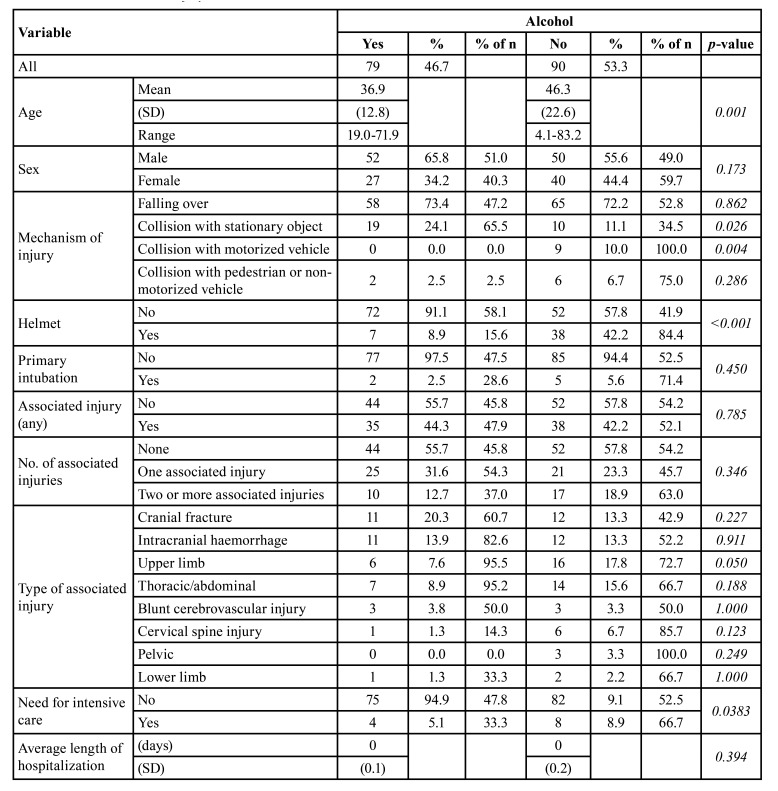
Association between injury-related variables and alcohol involvement.

Also, upper limb injuries were associated significantly with alcohol use (*p*=0.050).

Overall, alcohol involvement was significantly higher in e-scooter patients (*p*<0.001) (Table 2). Of all e-scooter patients, 88.9% (*n*=40) were under the influence of alcohol prior to the injury, compared with 31.5% (*n*=39) of patients in the bicycle group. Alcohol use reached RR of 9.11 for e-scooter use (95% CI 3.78 – 21.96).

Males were overrepresented in both groups, at 60.5% (*n*=75) in the bicycle group and at 60% (*n*=27) in the e-scooter group (Table 2). In addition, e-scooter patients were significantly younger than bicycle patients, with an average age of 29.8 (standard deviation [SD] ± 11.2) years compared with an average age of 46.3 8 (± 19.5) years in the bicycle group (*p*<0.001). The most frequent mechanism of injury in both groups was falling over. Collision with a stationary object was significantly more common among e-scooters (31.1%; *n*=14) than bicyclers (12.1%; *n*=15) (*p*=0.004). Roughly one-third (36.3%; *n*=45) of bicycle patients were wearing a helmet at the time of injury, while only one e-scooter (2.2%; *n*=1) was helmeted.

**Table 2:**
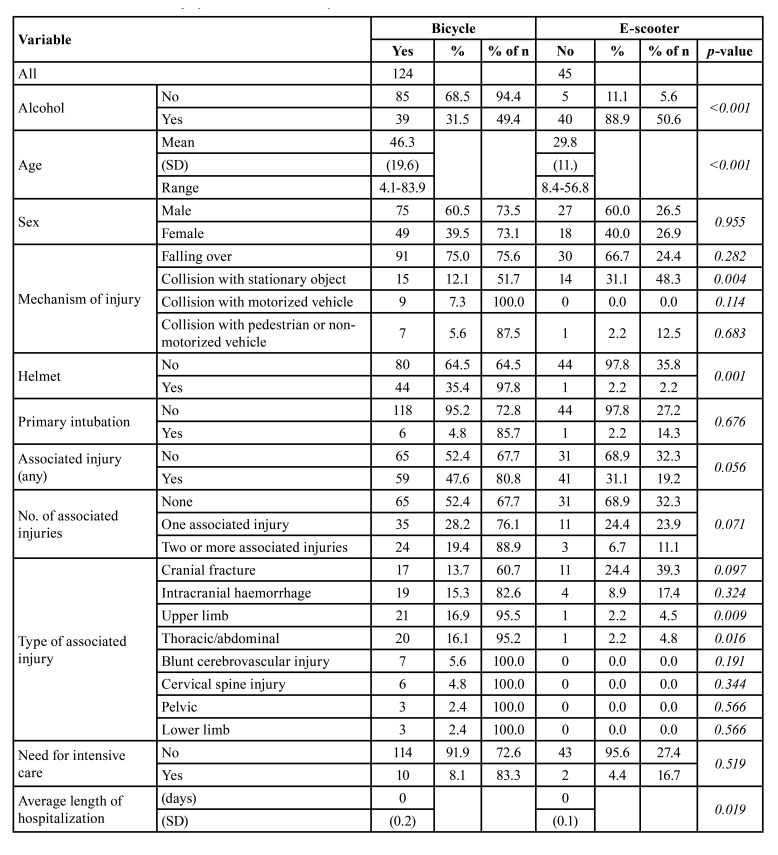
Association between injury-related variables in bicycle and electric scooter accidents.

Associated injuries (AIs) were common in both groups, as almost half (47.6%; *n*=59) of bicycle patients and almost one-third (31.1%; *n*=14) of e-scooter patients sustained AIs. In general, the prevailing types of AIs were cranial fracture (bicycle *n*=17, 13.7%, e-scooter *n*=11, 24.4%) and intracranial haemorrhage (bicycle *n*=19, 15.3%, e-scooter *n*=4, 8.9%). Statistical differences were found in upper limb injuries (*p*=0.009) and thoracic/abdominal injuries (*p*=0.016), with both of these injuries being more common among cyclists. Length of hospitalization was longer in cyclists (*p*=0.019).

In the multivariate analysis (Tables 3, Table 4), age (OR 0.93, CI 0.89-0.97, *p*=0.001), alcohol use (OR 17.85, CI 4.93-64.62, *p*<0.001), and cranial fractures (OR 10.15, CI 1.61-64.00, *p*=0.014) were independently associated with e-scooter accidents.

**Table 3:**
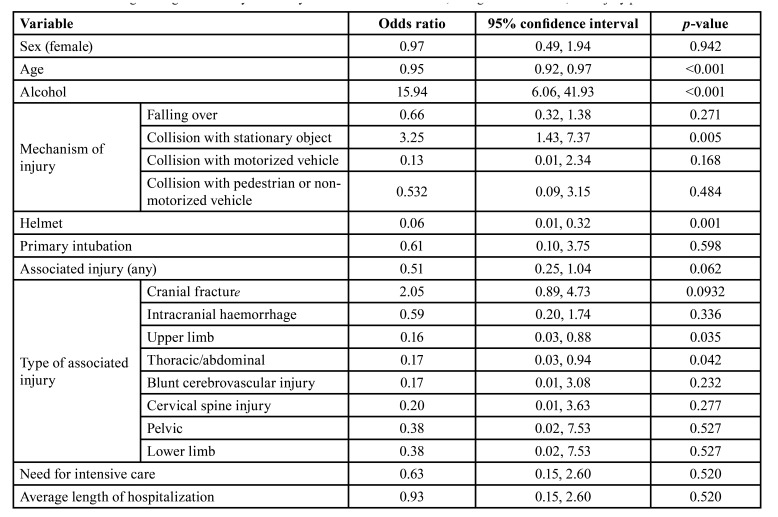
Univariate logistic regression analysis for bicycle and e-scooter accidents, background variables, and injury profile.

**Table 4:**
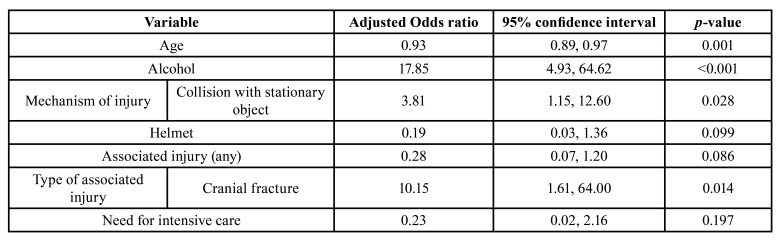
Multivariate logistic regression analysis for bicycle and e-scooter accidents, background variables, and injury profile.

**Figure 2:**
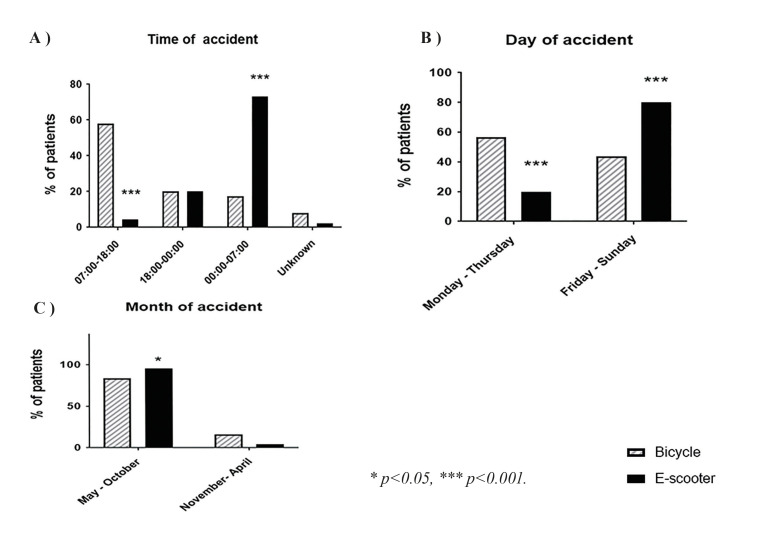
Time, weekday, and month of each bicycle- and e-scooter- related injury. a) Bicycle injuries took place mostly during 07:00 to 18:00 (*p*<0.001) whereas the majority of e-scooter injuries took place between midnight and 7 am (*p*<0.001). b) Virtually all e-scooter-related injuries took place during the weekends (*p*<0.001). c) E-scooter-related injuries were almost solely confined to the period between May and October, while bicycle-related injuries were slightly more evenly distributed throughout the year.

There was a significant difference between bicycle and e-scooter accidents regarding time of day and a significant difference regarding weekday of injury (Fig. 2). Of e-scooter accidents, 73.3% occurred during night-time, between midnight and 7 pm (*p*<0.001). In the bicycle group, injuries occurred more evenly throughout the day, with peak occurrence (58.1%, *n*=72) between 7 am and 6 pm. E-scooter accidents occurred also significantly more often on weekends (*p*<0.001) and during summer months (*p*=0.046).

## Discussion

This study clarified differences between the injury profiles related to bicycle and e-scooter accidents in a facial fracture patient population. Our hypothesis was confirmed. Alcohol use was strongly related to e-scooter accidents, and riding without a helmet characterized e-scooter drivers. Younger age, collision with a stationary object, and cranial fractures were independently associated with e-scooter riding. Also, compared with bicycle accidents, e-scooter-related injuries were more likely to occur on weekends, especially during the night-time.

E-scooter accident patients were on average 15 years younger than bicycle patients, with a mean age of 30 years. The youngest e-scooter patient was only 8 years old. In Finland, commercial e-scooter rideshare programmes have an age limit of 18 years, but with privately owned scooters there is no age requirement. One factor that might explain the age difference between e-scooter and bicycle patients is purpose of the travel. Bicycles are a popular mode of commute for working-aged people, whereas e-scooters are more often used for recreational purposes by socially active young adults (6,7). This notion is supported by our data since most e-scooter accidents occurred during weekend nights. Also, the use of rideshare e-scooters requires a specific mobile phone application, which younger people may find easier to use.

Alcohol use was common in both bicycle (31.5%) and e-scooter (88.9%) -related accidents, particularly among younger patients. We previously reported the prevalence of alcohol consumption in bicycle-related accidents facial fracture patients to be 30.5% (15). However, in a large multicentre study from Korea that included 19,842 bicycle accident patients, only 6.4% of patients were reportedly under the influence (16). Although the study did not specifically include facial fracture patients, the differences in alcohol prevalence indicate that driving under the influence seems to be country- and culture-dependent. The same trend can be observed in e-scooter patients. A study based on a Finnish population showed that 71% of e-scooter accidents resulting in traumatic brain injuries were related to alcohol use (10). According to a scoping review of the current literature, a much lower median of 26.5% of e-scooter accidents are related to alcohol (17). Although the high prevalence of alcohol involvement in bicycle- and e-scooter-related accidents may be partly due to cultural differences, it is noTable that alcohol seems to be a particular risk factor for head and facial injuries.

Alcohol is known to have negative effects on visuomotor performance, cognitive processing, reflexes, and reaction time. Impairment of these functions may also explain why alcohol was linked to such a large proportion of injuries in the present study, especially in the e-scooter group. Patients under the influence of alcohol are less likely to be able to break their fall with outstretching of the extremities, making the head and face the primary sites of energy transmission. In our study, cranial fractures were independently related to e-scooter injuries. E-scooter patients were more likely (24.4%) to suffer from cranial fractures than bicycle patients (13.7%), suggesting a more direct impact to the head without protective deceleration from other body parts. Compared with seated bicycle riders, standing e-scooter riders can achieve similar velocities while maintaining relatively high centres of gravity. This combination gives a falling e-scooter rider more momentum and even less time to react to the sudden loss of balance before impact, especially under the influence of alcohol. This notion is supported by the fact that only one e-scooter patient had a concomitant extremity fracture, whereas in the bicycle group 16.9% of patients had an associated upper limb fracture.

The lack of helmet use in both groups was significantly increased by alcohol use. At the time of the accident, one-third of bicycle patients and only one e-scooter patient was wearing a helmet. In a large registry-based study (*n*=76,032) by Scott *et al*., an even lower helmet compliance of 22.1% was reported among bicycle patients with head and neck injuries (18). In e-scooters, in accord with our results, Trivedi *et al*. found that none of the patients with e-scooter-related craniofacial injuries were wearing a helmet at the time of injury (11). Thus, the lack of helmet use is common in bicycle riders and very typical in e-scooter accidents despite the well-established protective role of helmets against head and facial injuries in bicycle-related accidents (19,20). Even though the direct association between craniofacial injuries and the protective role of helmets in e-scooter accidents is difficult to assess, since according to the literature the overall average helmet compliance of injured e-scooter riders is only 4.5%, our study emphasizes the importance of helmet use and the need for preemptive safety precautions (17). The lack of helmet use in the e-scooter population might partly explain the finding that cranial fractures were more than ten times more likely to be diagnosed in conjunction with e-scooter accidents than with bicycle accidents. It has also been shown that driving without a helmet is more prevalent among users of rideshare programmes than among privately owned bicycle or e-scooter users because of the lack of helmet availability (5). This is accentuated in e-scooter users since they are much more commonly a part of shared schemes than bicycle users.

Despite similar trauma mechanisms underlying these two modes of transport, achieving a high velocity with an e-scooter differs significantly from a bicycle. The acceleration of e-scooters is based on the turning of the throttle handle, an action requiring very limited coordination. This makes e-scooter riders driving under the influence of alcohol particularly susceptible to high-energy injuries. Interestingly, there were almost no collisions between e-scooter riders and other vehicles and pedestrians. This result is in line with a recent study by Stigson *et al*. who reported that 95.3% of e-scooter-related accidents did not involve collisions with a second party (21). This is most likely due to the observation that most e-scooter accidents took place during the evening or night-time when traffic is generally more tranquil than during the daytime. Another contributing factor is the number of simultaneous riders on each e-scooter board during transport. Although not separately presented in our data, trends in social behaviour are reflected in e-scooter riders often carrying passengers with them, which may increase susceptibility to injury. Further precautionary measures concerning e-scooter renting should be instigated. For example, the applications used to rent these vehicles could be developed to include algorithm-based verification stages to confirm that the rider is wearing a helmet prior to transport. Additionally, adding sensors to e-scooters that measure uneven distribution of weight on the board, i.e. when more than one rider is present, could be used to decrease e-scooter velocity as a safety measure.

Our study included only patients with facial fractures. Thus, the relatively low number of patients and the lack of non-facial fracture patients limit the drawing of broad conclusions and recommendations related to traffic behaviour in general. The retrospective study design may also result in underreporting of some injury-related parameters. 

## Conclusions

In the facial fracture population, e-scooter accidents differ from bicycle accidents, especially in terms of aetiological factors. Traffic safety can be improved by addressing drunken driving, by requiring the use of helmets, and by improving vehicle control by reducing driving speeds.
